# Diphtheria and Its Treatment with Antitoxin; with Report of Cases

**Published:** 1898-03

**Authors:** E. Brittain

**Affiliations:** Bremond, Texas


For the Texas Medical Journal.

### Diphtheria and Its Treatment With Antitoxin; With Report of Cases.

BY E. BRITTAIN, M. D., BREMOND, TEXAS.

I have treated sixteen (16) cases of diphtheria this year (1897) with Parke, Davis & Co.'s anti-diphtheritic serum, with only one death, and in this case serum was injected on fifth day—this being my first case treated with antitoxin. Only 1000 units were injected at first; no results of any consequence, and as the patient was gradually sinking, about sixteen hours later I injected 1500 units. A few minutes after this second dose almost a complete cast of trachea was expelled, or rather coughed up into the mouth and removed with forceps, as patient was too weak to cough it out. She then breathed better up to the time of her death, which occurred ten hours later. Nineteen cases were treated by others and myself without antitoxin, with seven (7) deaths. With one exception, all cases treated with and without antitoxin were under eight years of age, the majority from one to three years old; one case, a young lady, sixteen years old. Below I give a clinical report of some cases treated with antitoxin, but as it is unnecessary to go into details, will only report a few.

Case II.—Polish child; male; five years old. Was called to him on the fourth day of disease, the family having dismissed their attendant about one hour previously. Membrane extended almost completely over roof of the mouth and into the nose, and it may be unnecessary to say that the pharynx and larynx were also involved. Temperature, 103$\frac{4}{5}$ F. After washing back with soap and water and further cleansing with 5 per cent carbolized water, I injected 1500 units antitoxin subcutaneously on right side between vertebræ and scapula. Within twenty minutes temperature was reduced to 101$\frac{1}{2}$, and continued there for twelve hours. Although the general symptoms were much better, and most all of the membrane had disappeared, I was determined that not another case should die on my hands for lack of antitoxin, so I injected 1500 units again and prescribed the following:

℞ Hydrag. chor. corrosiv............gr. i

Tinct. ferri. muriat................ʒ iiiss

Syrupi...............................℥ j

Aquæ, q. s.................................℥ iii

M. Sig.: Teaspoonful in water every three hours.

Twenty-four hours later patient was much better. Membrane had all disappeared, but he was still very hoarse, and as a precautionary measure I injected 1500 units more, making a total of 4500 units for a five year old boy within forty-eight hours. Saw him again twenty-four hours later. Temperature about normal. Recovery uninterrupted, with the exception that a few days later paralysis of palate developed. This yielded in about three weeks to 40-drop doses syr. hypophos. comp. three times a day. Patient able to be up all the time after last injection.

Case III.—Boy 3 years old; temperature, 100⅕; pulse, 135; breathing heavy; had to sit up greater portion of night before I was called. The following morning gave 2 grain doses of calomel every two hours for five doses; injected antitoxin 1500 units a little below axilliary region; forty minutes later temperature was 99½. Twenty-four hours later, as calomel had acted well, I gave him iron and corrosive sublimate, as in case No. 2. At this time membrane had almost disappeared except in nose and posterior portion of phayrnx. Complete recovery within one week from first visit. No after effects.

Case IV.—Boy 4 years old; temperature, 104; pulse, 140. This was a case of naso-pharyngeal diphtheria, the membrane extending to outer edge of nose. Prescribed calomel in 2 grain doses for five doses and injected 1500 units antitoxin. Twenty hours later calomel had acted well, and I then prescribed iron and corrosive sub., as in other cases. Temperature, 102. Membrane had disappeared enough so that he could breathe through the nose. Injected 1500 units more antitoxin, and next day he was discharged cured. Four days later was called to see him again. Temperature, 103½; pulse rapid, but not counted, and scarlet rash over entire body. I prescribed for this as I would for any of the eruptive fevers, and he was again discharged cured in about one week.

A rash similar to the above appeared in two other cases only of the total number treated. In one case the rash appeared two weeks after a single injection of 1500 units. Patient had been out of bed for a week before it appeared.

Now for synopsis of treatment: First, inject antitoxin from 500 to 2000 units, as the case demands; give a course of calomel in from 1 to 5 grain doses every two hours for four or five doses; after this has acted well, give whiskey freely, corrosive sub. and iron as above and you will rarely need to give a second course of calomel.

Locally, use first Bovinine pure in spray, then, after thoroughly washing, spray with warm water; spray throat with Peroxid. Hydrogen pure, or diluted, or throat may be swabbed with this or similar solutions every three hours.

For systemic or local treatment act as your judgment dictates. Only use a sufficient quantity of antitoxin injected sub-cutaneously, either in back between scapulæ or immediately under axillary region, and you will rarely, if ever, have a death to record from diphtheria.

I have used only Parke, Davis & Co.'s Anti-Diphtheritic Serum and as results have been so satisfactory will continue to use it.

